# 26.48% efficient and stable FAPbI_3_ perovskite solar cells employing SrCu_2_O_2_ as hole transport layer

**DOI:** 10.1039/d2ra06535e

**Published:** 2023-01-11

**Authors:** Muhammad Noman, Muhammad Shahzaib, Shayan Tariq Jan, Syed Nasir Shah, Adnan Daud Khan

**Affiliations:** a U.S.-Pakistan Center for Advanced Studies in Energy, University of Engineering & Technology Peshawar Pakistan muhammad.noman@uetpeshawar.edu.pk; b Department of Energy Engineering, Faculty of Mechanical and Aeronautical Engineering, University of Engineering and Technology Taxila 47080 Rawalpindi Pakistan; c Department of Energy Technology, University of Technology Nowshera Pakistan

## Abstract

In general, formamidinium lead tri-iodide (FAPbI_3_) based perovskite solar cells are more stable than their methylammonium lead tri-iodide (MAPbI_3_) counterparts. However, when it comes to power conversion efficiency (PCE), MAPbI_3_ solar cells are far better. This work aimed to enhance the power conversion efficiency of FAPbI_3_ solar cells without compromising their thermal stability. The numerical analysis of 6 different proposed structures with 2 carbon based electron transport materials (C_60_, PCBM) and 3 copper based hole transport materials (SrCu_2_O_2_, CuSCN, CuSbS_2_) is performed using SCAPS-1D software. The parameters are used from various theoretical and experimental published works. In order to investigate the performance of each proposed structure, the defect density, layer thickness and doping concentration of the absorber layer, electron transport layer (ETL) and hole transport layer (HTL) are varied, and optimized parameters are enumerated. The best simulation result having PCE of 26.48% is achieved with 1.25 V open circuit voltage (*V*_OC_), 23.51 mA cm^−2^ short circuit current (*J*_SC_) and 89.5% fill factor (FF) for FTO/PCBM/FAPbI_3_/SrCu_2_O_2_/Au. The proposed structure also showed good thermal stability at 300 K. Moreover, the effects of the different charge transport layer on the energy band alignment, electric field, recombination and IV characteristics are also investigated in detail.

## Abstract 2

The effect of carbon and copper based charge transport layers on the behavior and performance of FAPbI_3_ perovskite solar cells was investigated with SrCu_2_O_2_/FAPbI_3_/PCBM structure outperforming others and achieving 26.48% efficiency.

## Graphical abstract

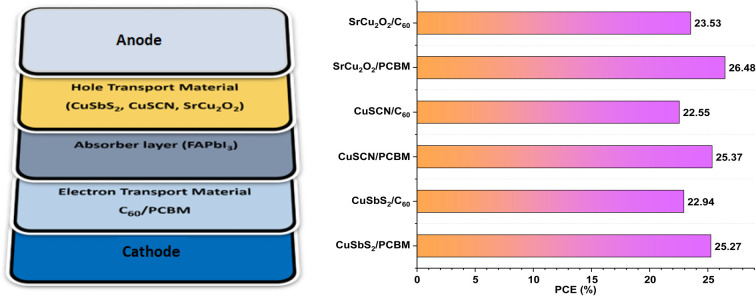

## 1 Introduction

Since the last decade, technological developments in the area of organic–inorganic solar cells have transformed the research landscape in the quest for a reliable and practical alternative to currently existing energy sources. Perovskite solar cells have achieved a huge leap in their power conversion efficiency (PCE) within a short time due to their facile manufacturing process and relatively low operating cost.^1^ Due to the outstanding results achieved so far, it has gained huge attention from researchers around the world.^2^ One of the most significant advantages and attributes of hybrid perovskite solar cells is their skyrocketing reputable power conversion efficiency of more than 25%, which is due to their wide band gap, high absorption coefficient, low range of exciton binding energy, low surface recombination rate, high mobility of charge carriers, and long diffusion length.^3,4^ As a result of its cost effectiveness and enhanced performance, the perovskite based solar cell has become one of the most promising PV technologies.^5–7^

The first ever expedition to the perovskite solar cell was proposed in 2009 by Kojima *et al.* utilizing iodine (I^−^) and bromine (Br^−^) as the halide materials and reported PCE up to 3.81%.^8^ After two years, a perovskite solar cell with a size of 2–3 nm nanocrystal attained a power (PCE) of 6.54% in 2011. In the year 2013, PCE hit a record high of 16.2%.^9^ In the same year a layer modification of TiO_2_ yielded PCE of 19.3% due to improved electrical and optical characteristics.^10^ In 2017, PCE of 20.1% was claimed by the Korean researchers Y. W. S. and N. J. H. *et al.* While, after a decade from its first ever reported structure, PCE of 23% was achieved in the year 2019 using methylammonium lead tri-iodide (MAPbI_3_) as a perovskite absorbing material.^11^ Numerous researchers contributed to improve the performance of perovskite solar cells (PSCs) during the last decade. However, the efficiencies of perovskite solar cells have not yet attained the maximum theoretical limit of Shockley–Queisser, which is approximately 31.4%.^12^

Researchers have attempted to improve the PV performance of MAPbI_3_ based perovskite solar cell using different techniques.^13–15^ However, due to degradation and moisture sensitivity issues it has raised some serious device stability concerns.^16^ In contrast to the MAPbI_3_, formamidinium lead tri-iodide (HC(NH_2_)_2_PbI_3_ or FAPbI_3_) based perovskite solar cell has attained higher thermal stability.^17^ Furthermore, FAPbI_3_ has an energy band gap of 1.48 eV, which is more suitable for capturing the solar spectrum.^18–21^

Charge transport layers (ETL & HTL) have a significant effect to boost the PCE during the photogeneration of electron (e^−^) and the extraction of hole (h^+^) from the absorber layer. To achieve better PSC performance, a suitable combination of available hole transporting material (HTL) and electron transporting material (ETL) should be investigated along with the absorber layer. The most extensively used HTL in latest literature is spiro OMeTAD which has an organic nature.^22^ By incorporating 4-*tert*-butylpyridine (TBP) and bis(trifluoro methane) sulfonamide lithium salt (Li-TFSI) the hole mobility and conductivity of spiro OMeTAD is accelerated.^23^ Though they both support to improve polarity but, they come with some major drawbacks, such as dissolving the absorber (TBP) and encouraging oxidation in spiro OMeTAD (Li-TFSI), which degrades the absorber.^24,25^ Another widely used organic HTL is PEDOT:PSS, which is chemically unstable. Furthermore, due to its acidic nature, the cell is prone to corrosion.^26^ Because organic HTLs are significantly more expensive, they must be replaced with highly efficient, long-term stable, and cost-effective inorganic alternatives.^26^

In this work, we have extensively studied the FAPbI_3_ as an absorber layer with three different copper based HTMs, copper thiocyanate (CuSCN), copper antimony sulfide (CuSbS_2_) and strontium cuprate (SrCu_2_O_2_) along with carbon based ETL (C_60_ & PCBM). A numerical comparison of three distinct HTL is illustrated in terms of open circuit voltage, short circuit current density, fill factor and power conversion efficiency. Analysis has shown efficient results for HTL/SrCu_2_O_2_ with PCBM as electron transport layer in comparison to C_60_. The impact on electrical responses of structure has been analyzed by varying absorber layer thickness, absorber defect density, CTL thickness and HTL & ETL doping concentration. Finally, we have studied the effect of temperature on the stability and power conversion efficiency. Employing these factors, optimum solar cell parameters are identified, and a significant improvement in power conversion efficiency is attained.

## 2 Device modeling and simulation

**Fig. 1:**
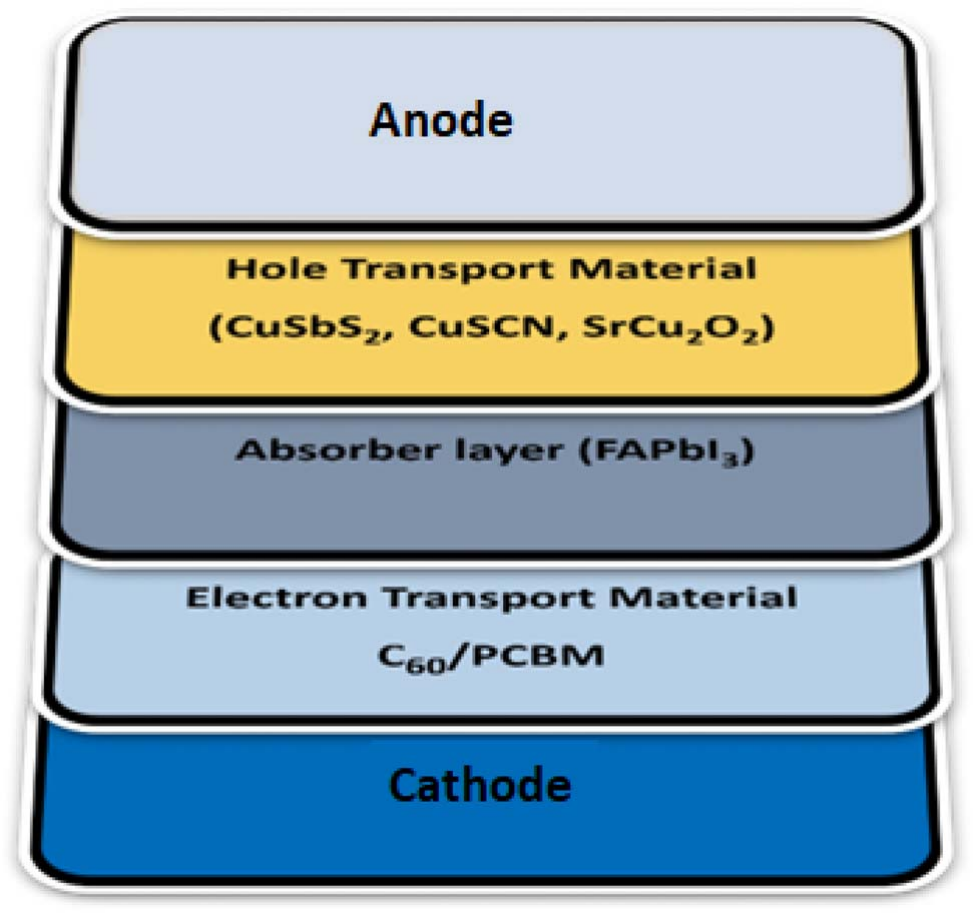
Schematic visualization of device architecture.

Solar cell capacitance simulator software SCAPS (ver.3.3.10) under AM1.5G (100 mW cm^−2^) solar spectrum with 300 K temperature has been used as a simulation tool to observe the electrical responses and impact on the perovskite structure. It is a one-dimensional opto-electrical simulator tool which can calculate the response of multi semiconductor layers. Its working principle is based on continuity and Poisson equations.^27,28^ Our simulated n–i–p structure composed of FTO/C_60_, PCBM/FAPbI_3_/CuSbS_2_, SrCu_2_O_2_ & CuSCN/Au, is shown in Fig. 1. To ensure efficient device performance, the thickness of the absorber layer, HTL, and ETL is tuned over a wide range to attain optimum values. FAPbI_3_ serves as the light absorber layer in all device configurations, sandwiched between the charge transport layers (ETL and HTL). The parameters required in the simulation such as thickness of each layer, e^−^ and h^+^ mobility, bandgap of materials, effective density of states and defect density are precisely taken from different experimental and theoretical published work, see Table 1. The thermal velocity for e^−^ and h^+^ are set to 1 × 10^7^ cm s^−1^. The value of work function (front contact – FTO) and electrode (back contact – Au) are considered as 4.04 eV and 5.9 eV respectively.

**Table 1:** Input parameters for optoelectronic simulation

Parameters	SrCu_2_O_2_ (ref. 29)	CuSbS_2_ (ref. 30)	CuSCN^31,32^	FAPbI_3_ (ref. 4 and 33)	C_60_ (ref. 34)	PCBM^35^
Thickness (nm)	150	150	150	300	150	150
Band gap (eV)	3.3	1.58	3.2	1.51	1.7	2
e^−^ affinity (eV)	2.2	4.2	1.9	4	3.9	4.2
Permittivity	9.77	14.6	10	6.6	4.2	3.9
CB effective density of states (cm^−3^)	2.2 × 10^18^	2.0 × 10^18^	2.2 × 10^19^	1.2 × 10^19^	8 × 10^19^	2.5 × 10^21^
VB effective density of states (cm^−3^)	1.8 × 10^19^	1.0 × 10^19^	1.8 × 10^19^	2.9 × 10^18^	8 × 10^19^	2.5 × 10^21^
Mobility of e^−^	0.1	49	2 × 10^−4^	2.7	8 × 10^−2^	0.2
Mobility of h^+^	0.46	49	1 × 10^−2^	1.8	3.5 × 10^−3^	0.2
Density of n-type doping	0	0	0	1 × 10^16^	2.6 × 10^17^	2.9 × 10^17^
Density of p-type doping	3.6 × 10^18^	1 × 10^18^	1 × 10^18^	1 × 10^16^	0	0
Density of defects	1 × 10^15^	1 × 10^15^	1 × 10^15^	1 × 10^14^	1 × 10^15^	1 × 10^15^
Interface defects	1 × 10^12^	1 × 10^12^	1 × 10^12^		1 × 10^12^	1 × 10^12^

## 3 Result and discussion

The primary focus of this research was to investigate the effect of different charge transport materials on the performance of FAPbI_3_ solar cell. In this regard, the energy band alignment, electric field, recombination and IV are first investigated for each structure. For optimized layers, the thickness of the absorber layer, ETL and HTL is varied initially. After identifying the optimized thickness, the doping concentration of absorber and charge transport layers are optimized. Influence of defect density was observed after getting optimized values of the configuration. Finally, the effect of temperature on the performance of device configuration is observed.

### 3.1 Energy band alignment

The energy band alignment of the charge transport material (HTL & ETL) with the perovskite material plays a very important role in the performance of the PSC. For efficient separation of electrons from the perovskite material the conduction band of ETL and PSC should align with minimum offset, while their valence band should have a large offset. If the valence bands are near then there is a probability that holes will flow to the ETL which would cause recombination. Similarly for separation of holes from the perovskite material the valence band of HTL and PSC should align, while their conduction band should have a large offset. If the conduction bands are near then there is a probability that electrons will flow to the HTL which would cause recombination.

**Fig. 2:**
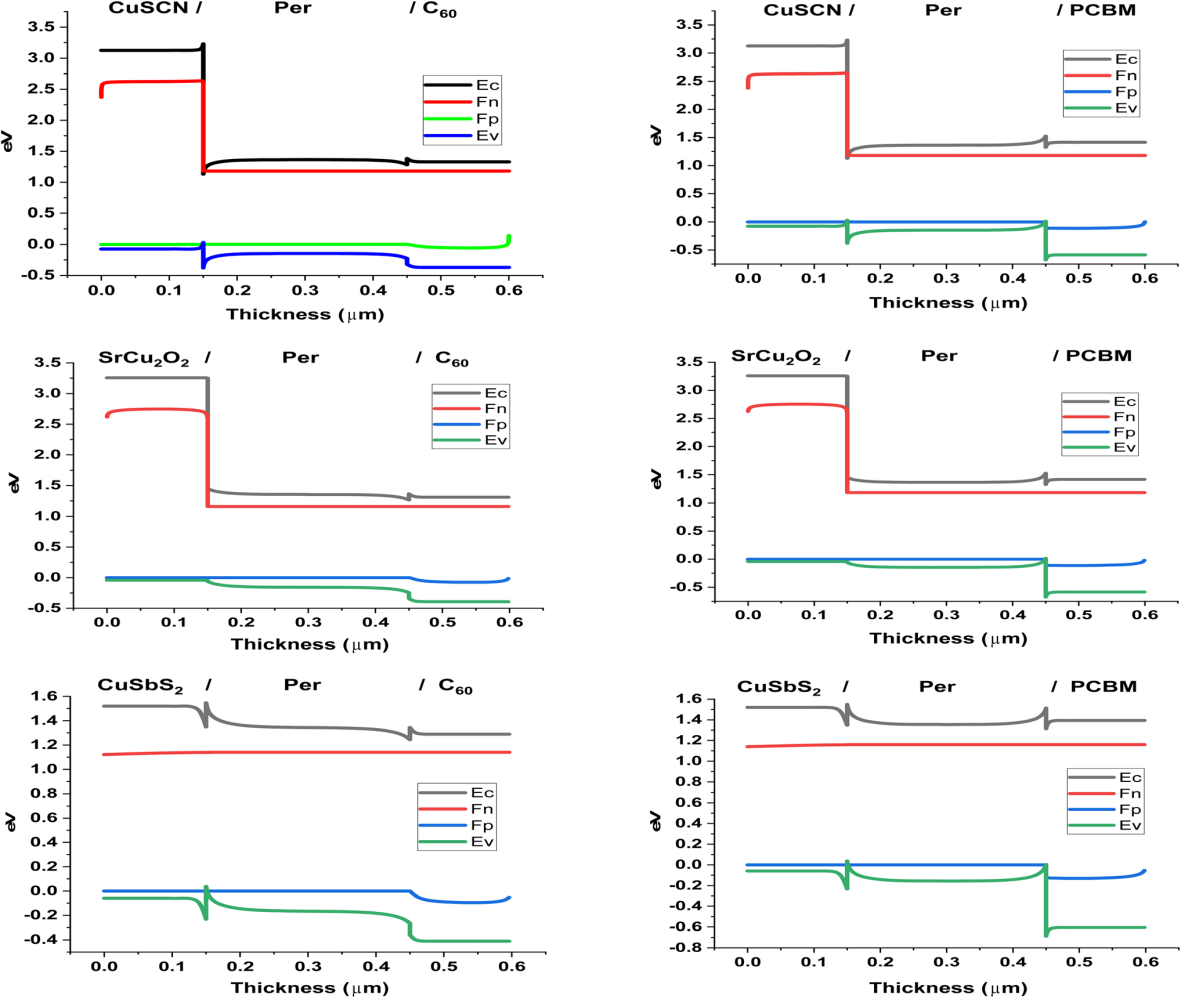
Energy band alignment of PSC layers.

Fig. 2 shows the energy band alignment of the different structures while Table 2 presents conduction band offset (CBO) and valence band offset (VBO) of the CTL with the perovskite material. SrCu_2_O_2_ makes perfect band alignment as its valence band has minimum offset of −0.01 eV with valence band of perovskite and large conduction band offset of 1.8 eV. The valence band of CuSbS_2_ forms a spike at the heterojunction of 0.27 eV which may increase the flow of holes but due to its small conduction band offset of −0.2 eV, electrons may also cross over, contributing in recombination. While the CuSCN forms a cliff of −0.41 at the interface which causes large hurdles for the holes to cross over, hence increasing recombination and reducing performance.

**Table 2:** CBO and VBO at interfaces

Interface	CBO (eV)	VBO (eV)
**ETL/perovskite**		
C_60_/perovskite	0.1	0.09
PCBM/perovskite	−0.2	0.69

**Perovskite/HTL**		
Perovskite/SrCu_2_O_2_	1.8	−0.01
Perovskite/CuSbS_2_	−0.2	0.27
Perovskite/CuSCN	2.1	−0.41

Similarly, for the ETL energy band alignment with the perovskite, both materials form small conduction band offsets. C_60_ forms a small spike of 0.1 eV while PCBM forms a small cliff of −0.2 eV. The PCBM outperforms the C_60_ due to the larger valence band offset of 0.69 eV it forms with the perovskite, which blocks the crossing over of the holes. Due to C_60_ small VBO of 0.09 eV, holes also flow to the ETL which causes increase in recombination.

### 3.2 Electric field

**Fig. 3:**
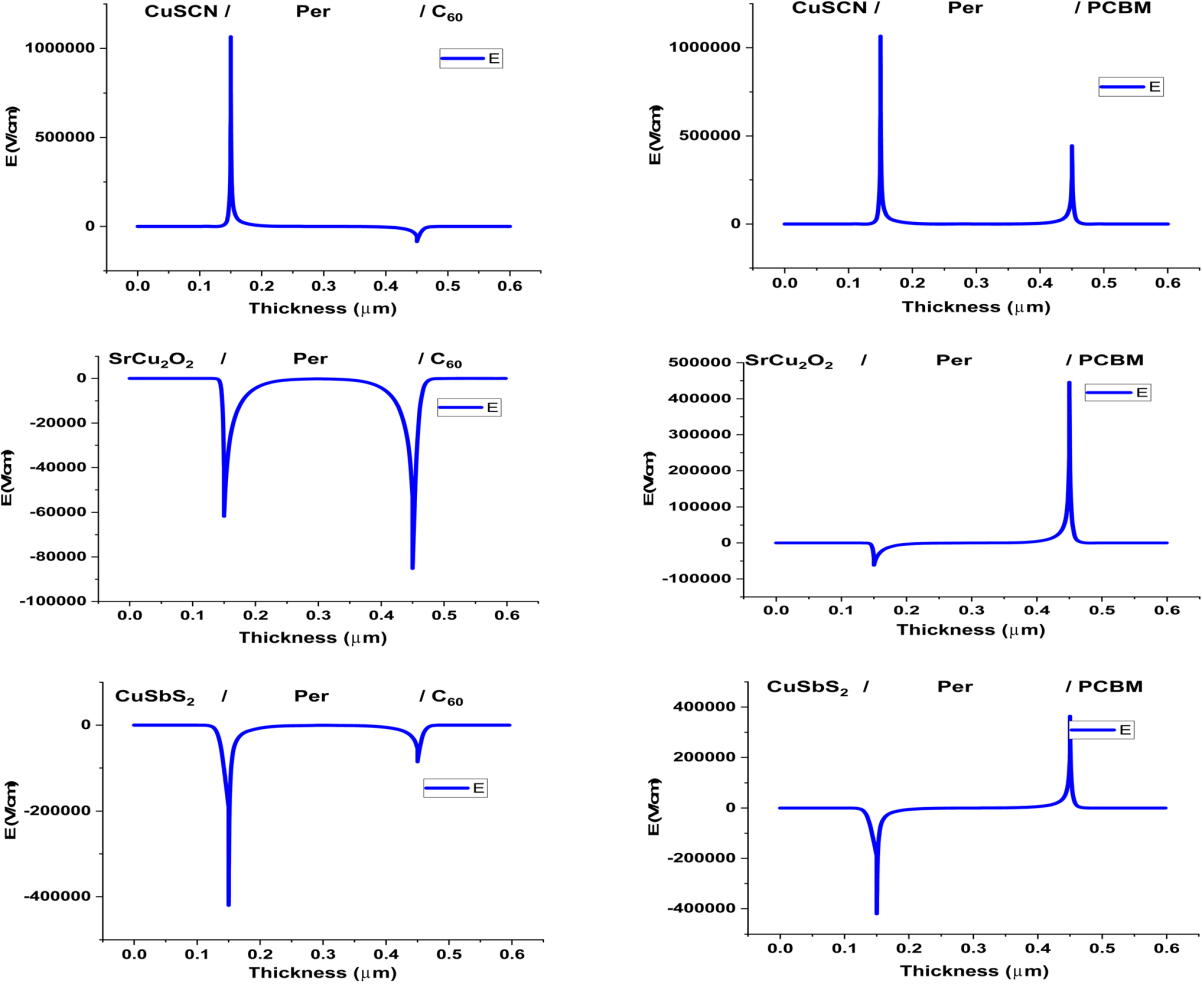
Electric field at PSC layer interfaces.

The electric field produced by the charge transport material with the perovskite helps in separating the charge carriers in the perovskite. High electric field at the interface will separate more charge carriers as its influence will be deeper in the perovskite material. Fig. 3 shows the electric field produced by the CTM in the different PSC structures. A positive band offset (spike) increases the built in potential at the interface while a negative band offset (cliff) reduces the built in potential. It can be seen that in the ETL the C_60_ produces a negative electric field due to the positive conduction band offset (0.1 eV). While the PCBM produces a positive electric field due to the large negative conduction band offset (−0.2 eV). The negative conduction band offset not only reduces the built in potential but the cliff also blocks the flow of electrons to the ETL. The electrons accumulate at the heterojunction and produce its own electric field, opposite to the heterojunction's; thus, a positive electric field is obtained.

Similarly, for the HTL the CuSbS_2_ produces the negative electric field due to its large positive valence band offset (0.27 eV). The electric field by SrCu_2_O_2_ is also negative because the valence band offset is very minute (−0.01 eV). While CuSCN produced a positive electric field due to its large negative valence band offset (−0.41 eV) which blocks the flow of holes to the HTL.

### 3.3 Recombination

**Fig. 4:**
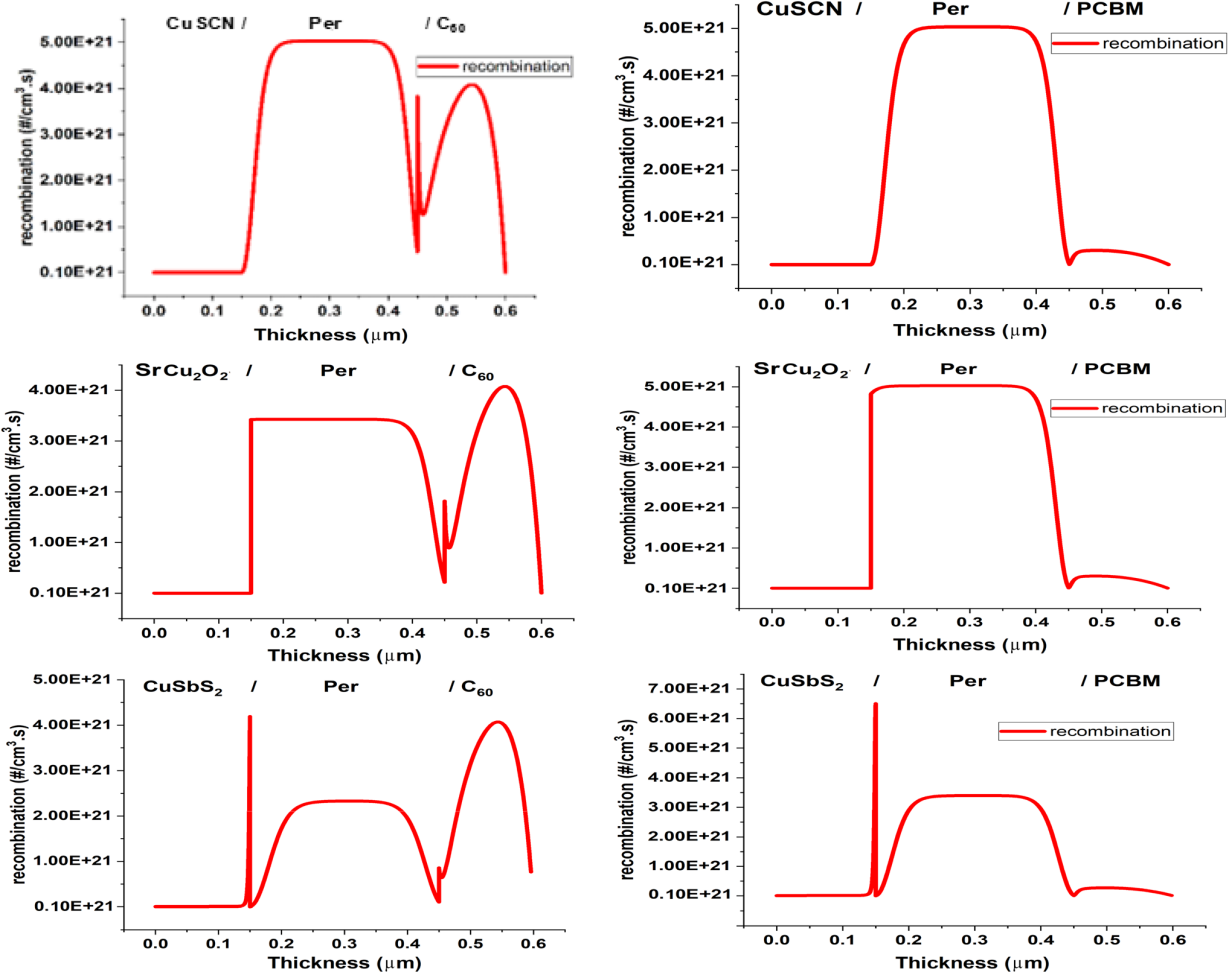
Recombination in PSC structures.

Recombination is the recombining of the photogenerated charge carriers. Ideal PSC should have minimum recombination at the CTL/perovskite interface so that maximum carriers can be collected. Fig. 4 shows the recombination occurring in the PSC structures. The PCBM makes an acceptable CBO of −0.2 eV and large VBO with the perovskite material ensuring the smooth flow of electrons to the ETL where they are transported to the electrode for collection. The ETL cause minimum recombination at the heterojunction. As for C_60_, due to the low valence band offset, holes also flow from the perovskite to the ETL. This causes an increase in recombination in the ETL and reduces performance.

For the HTL SrCu_2_O_2_ forms the best energy band alignment with small VBO and large CBO. This ensures the smooth flow of the holes to the HTL while the electrons are blocked, causing minimum recombination at the heterojunction. The CuSbS_2_ forms small VBO and CBO which causes both holes and electrons to flow to the HTL. This causes an increase in recombination in it and reduces performance. While CuSCN forms a large VBO which blocks the flow of holes to the HTL and increases recombination.

### 3.4 IV characteristics

**Fig. 5:**
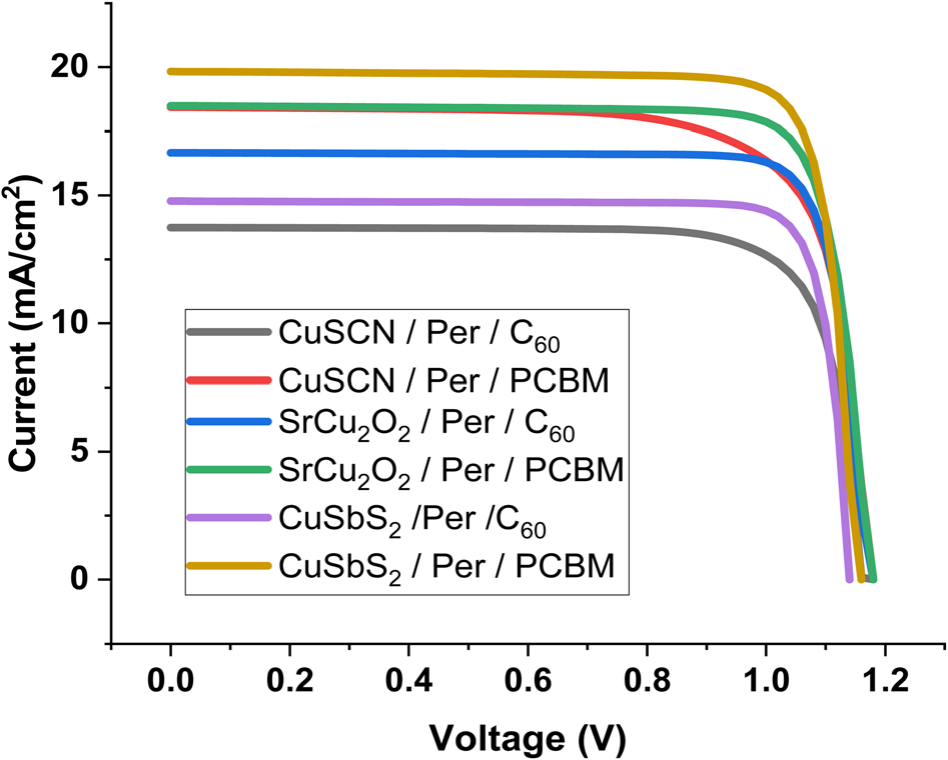
IV of PSC structures.

Fig. 5 shows the IV characteristics of the six different structures. From the above-mentioned factors, it can be seen that the PCBM structures outperform the C_60_ structures. As less recombination occurs in PCBM, more current is produced. This leads to more efficiency and better performance.

### 3.5 Effect of absorber layer thickness and defect density

**Fig. 6:**
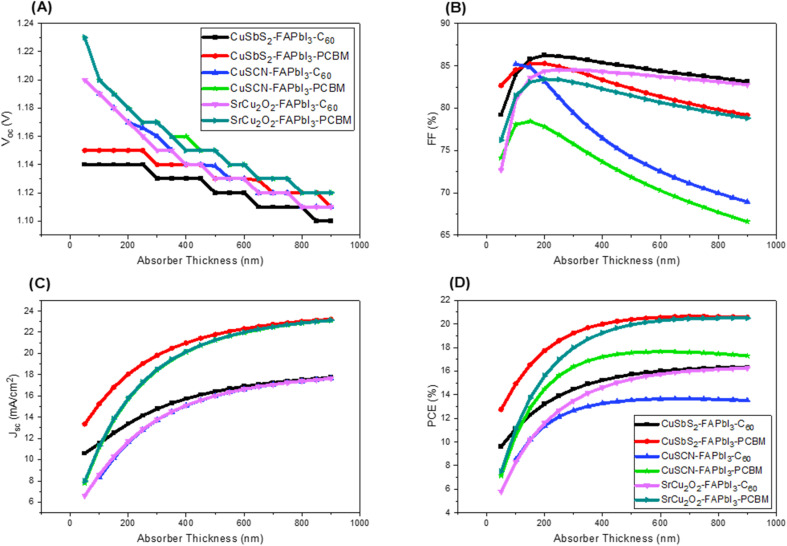
Impact of varying absorber layer thickness on (A) *V*_oc_ (B) FF (C) *J*_sc_ (D) PCE.

Absorber layer thickness is one of the major parameters in the configuration of solar cell. The thickness variation causes significant effect on the device output *V*_OC_, *J*_SC_, FF and PCE. In order to optimize the thickness of the absorber layer, it has been varied from 50 nm to 900 nm with an increment of 50 nm. Fig. 6 shows the effect of varying absorber thickness on device output characteristics. The detailed comparative analysis of device output characteristics with and without optimized absorber layer thickness is summarized in Table 3. It can be seen that as we start increasing the thickness of absorber layer from its initial value, the PCE increases. This is due to the fact that increase in absorber layer thickness provides more area for light to get absorbed which elevates the *J*_SC_ and hence PCE increases. However, after certain increase in PCE further increase in absorber layer thickness may not result in the improvement of output power. If we increase the absorber layer thickness further, the PCE starts to decrease. This is caused due to increase number of traps and majority of the excess carriers are not reaching the electrodes.^36^

**Table 3:** Comparative analysis of device output characteristics for optimized & non-optimized absorber thickness

Structure	Status	Absorber thickness (nm)	*V*_OC_ (V)	*J*_SC_ (mA cm^−2^)	FF%	PCE%
CuSbS_2_/C_60_	Initial	300	1.13	14.78	85.91	14.47
	Optimized	800	1.11	17.51	83.57	16.28
CuSCN/C_60_	Initial	300	1.16	13.74	79.42	12.67
	Optimized	550	1.13	16.33	73.32	13.6
SrCu_2_O_2_/C_60_	Initial	300	1.15	13.77	84.52	13.49
	Optimized	800	1.11	17.39	83.12	16.16
CuSbS_2_/PCBM	Initial	300	1.14	19.82	84.46	19.22
	Optimized	650	1.13	22.54	80.94	20.62
CuSCN/PCBM	Initial	300	1.17	18.43	75.75	16.36
	Optimized	550	1.14	21.64	71.03	17.63
SrCu_2_O_2_/PCBM	Initial	300	1.17	18.49	83.06	17.98
	Optimized	700	1.13	22.51	80.01	20.45

As far as the effect on *V*_OC_ is concerned, the graph in Fig. 2(A) shows significant dip as absorber thickness is increased. As the absorber thickness increases, carrier concentration increases and hence resulting in higher photo-generated current. However, due to high recombination rate and higher dark current *V*_OC_ significantly decreased.

**Fig. 7:**
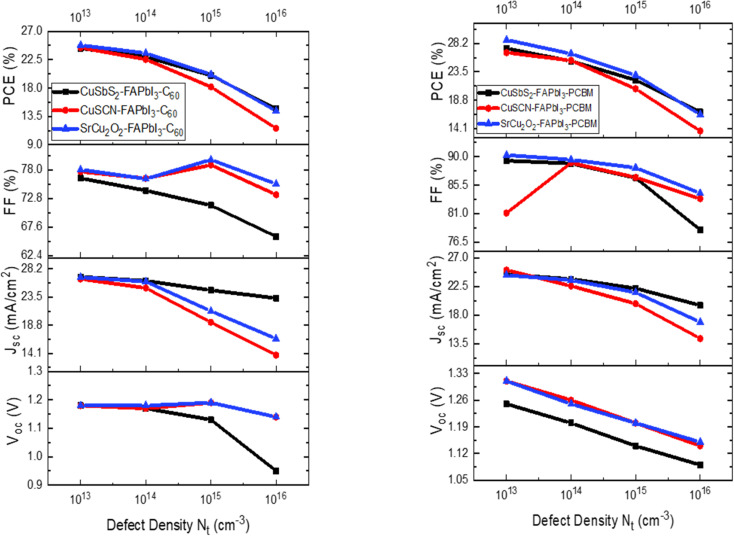
Influence of defect density (*N*_t_) on device output characteristics.

The absorber defect density (*N*_t_) has a significant effect on the performance of the solar cell and the optimized thickness as it traps the photo-generated carriers inside the trap levels. This reduces the carrier life time which in turn reduces the absorption length and increases the recombination.^38,39^ In order to investigate the effect of absorber *N*_t_, the energetic distribution of the defects is kept as Gaussian distribution. The literature on different experimental findings showed better output performance for perovskite of highly crystalline nature at lower *N*_t_. From the results in Fig. 7 it can be seen that at lower *N*_t_ (1 × 10^11^ and 1 × 10^12^), there is a negligible effect on the performance of the solar cell. However, when it increased from 1 × 10^13^ to 1 × 10^16^, significant reduction in *J*_SC_ and PCE is observed. In order to achieve an optimum and more practical results, *N*_t_ is chosen at 1 × 10^14^ throughout this work.^40^

Table 4 depicts the optimized thickness and output parameters on different defect densities for the proposed device structures.

**Table 4:** Optimized solar cell parameters for different *N*_t_ of proposed structures

*N*_t_	Thickness (nm)	Doping concentration	*V*_OC_ (V)	*J*_SC_ (mA cm^−2^)	FF (%)	PCE (%)
1 × 10^13^	900	1 × 10^18^	1.31	24.29	90.24	28.78
1 × 10^14^	700	1 × 10^18^	1.25	23.51	89.5	26.48
1 × 10^15^	550	1 × 10^18^	1.2	21.56	88.23	22.93
1 × 10^16^	400	1 × 10^18^	1.15	16.89	84.22	16.41
1 × 10^17^	300	1 × 10^18^	1.18	10.9	76.15	9.88
1 × 10^18^	100	1 × 10^18^	1.0	7.65	60.37	4.65

### 3.6 Effect of CTL thickness

**Fig. 8:**
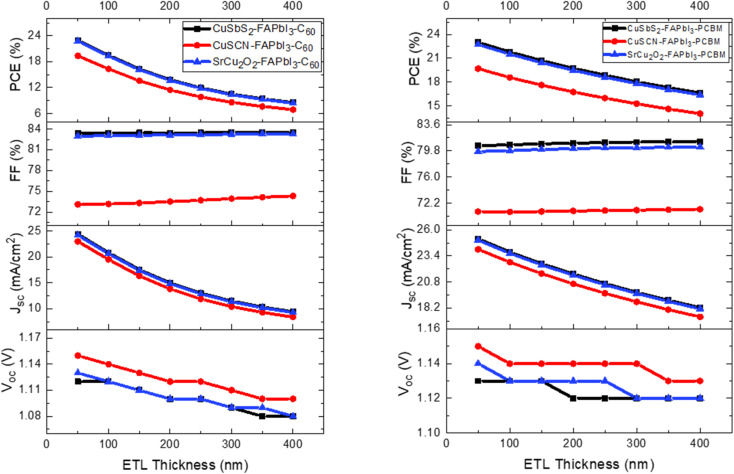
Influence of ETL thickness on device output characteristics.

**Fig. 9:**
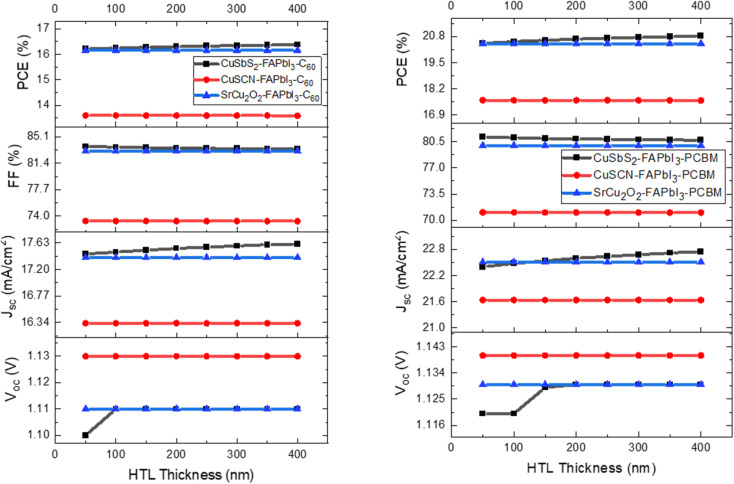
Influence of HTL thickness on device output characteristics.

The variation in CTL thickness for proposed device structures has been analyzed in detail by observing their effect on the device output characteristics. The CTL thickness is varied from 50 nm to 400 nm with an increment of 50 nm. Fig. 8 shows the effect of varying ETL thickness and Fig. 9 shows the effect of varying HTL thickness on the device output characteristics. The increase in CTL thickness leads to increase in series resistance which makes it difficult for the charge ions to reach the electrodes and hence recombination occurs. Besides transporting the photo generated charge carriers, the other function of the CTL is to act as a buffer layer between the organic perovskite layer and metal electrodes as direct contact between the two layers produce high series resistance. Too thin CTL are not able to provide adequate separation between the two layers. Therefore, in this study, the optimum thickness for ETL and HTL are chosen at 100 nm as further increase leads to reduction in PCE.

### 3.7 Effect of absorber doping concentration

**Fig. 10:**
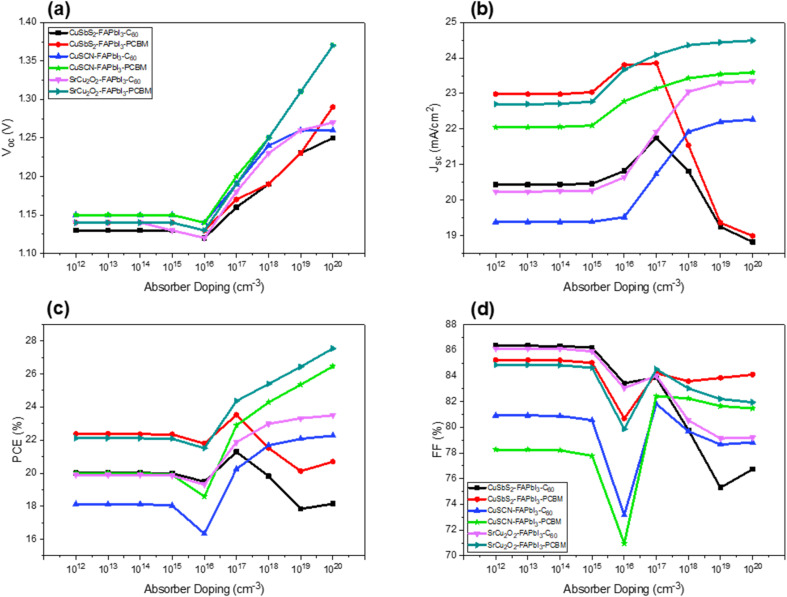
Influence of absorber doping concentration on device output characteristics (a) *V*_oc_ (b) *J*_sc_ (c) PCE (d) FF.

The output performance of the perovskite solar cells is highly influenced by the quality and structure of the absorber layer. Doping concentration and defect density plays a significant role in obtaining effective device outcomes. To investigate the effect of doping concentration on the output performance of the solar cell, the acceptor doping concentration of the absorber layer is varied from 1 × 10^12^ to 1 × 10^20^ cm^−3^. The optimized thickness and doping concentration in the remaining layers are kept constant. It is quite evident from the Fig. 10 that the doping concentration from 1 × 10^12^ to 1 × 10^15^ cm^−3^ has a very minute effect on the output performance of the cell. However, from 1 × 10^16^ cm^−3^ and onwards the *V*_OC_ and PCE increased significantly due to the decrease in reverse saturation current and increase in charge carrier concentration. An increased efficiency of up to 4% can be seen for each structure with an increase of doping concentration. A decrease in *J*_SC_ and PCE can be observed in CuSbS_2_ structures after doping concentration increased to 1 × 10^17^ cm^−3^. Heavy doping leads to increased recombination because of too many scattering centers.^37^ Furthermore, it causes the perovskite to change from semiconductor to metallic nature, which has a significant effect on the charge carrier transport mechanism. From the results it can be concluded that apart from CuSbS_2_ the optimized doping concentration for all the proposed structures is achieved at 1 × 10^18^ cm^−3^.

### 3.8 Effect of CTL doping

**Fig. 11:**
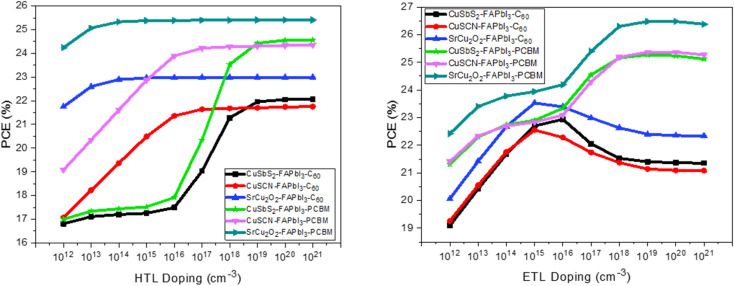
Influence of CTL doping on power conversion efficiency of the device.

To investigate the doping effect of CTL on the performance of perovskite solar cell, the doping levels are varied from 1 × 10^12^ to 1 × 10^20^ cm^−3^. The acceptor concentration (*N*_A_) is increased for HTM while donor concentration (*N*_D_) is increased for ETM. The results are analyzed to find the optimized parameters for proposed structures. The results in Fig. 11 shows that by increasing *N*_A_ from 1 × 10^16^ cm^−3^ to 1 × 10^18^ cm^−3^, a significant increase in PCE is observed for CuSbS_2_ and CuSCN. While in case of SrCu_2_O_2_, it is comparatively less. However, after 1 × 10^18^ cm^−3^ the saturation point is achieved. In this work we choose 1 × 10^20^ cm^−3^ value of doping for CuSbS_2_ and CuSCN to attain efficient results.

The behavior of the perovskite solar cell is different for both C_60_ and PCBM as evident from Fig. 11. The structures with C_60_ displayed better PCE as compared to PCBM when the doping concentration is increased up to 1 × 10^15^ cm^−3^. The structures with PCBM showed a steady increase in PCE initially when the doping concentration is increased till 1 × 10^15^ cm^−3^. However, further increased in doping concentration showed significant rise in PCE. Finally, saturation occurred after 1 × 10^19^ cm^−3^ due to the Moss–Burstein effect.^41^

The optimized doping for the proposed structures with C_60_ is found at 1 × 10^15^ cm^−3^ while, for PCBM it is found at 1 × 10^19^ cm^−3^. These doping levels give maximum PCE of 23.53% and 26.48% respectively. Table 5 depicts the solar cell parameters with possible combination of HTM and ETM. The maximum PCE of 26.48% is achieved for FTO/PCBM/FAPbI_3_/SrCu_2_O_2_/Au.

**Table 5:** Optimized solar cell parameters along with device output characteristics for proposed structures

HTM	ETM	Optimized doping of HTM (cm^−3^)	Optimized doping of ETM (cm^−3^)	*V*_OC_ (V)	*J*_SC_ (mA cm^−2^)	FF (%)	PCE (%)
CuSbS_2_	PCBM	10^20^	10^19^	1.2	23.67	88.91	25.27
	C_60_	10^20^	10^16^	1.17	26.20	74.25	22.94
CuSCN	PCBM	10^19^	10^19^	1.26	22.59	89.01	25.37
	C_60_	10^20^	10^15^	1.17	25.00	76.46	22.55
SrCu_2_O_2_	PCBM	10^18^	10^19^	1.25	23.51	89.5	26.48
	C_60_	10^18^	10^15^	1.18	26.08	76.38	23.53

### 3.9 Effect of temperature on solar cell performance

**Fig. 12:**
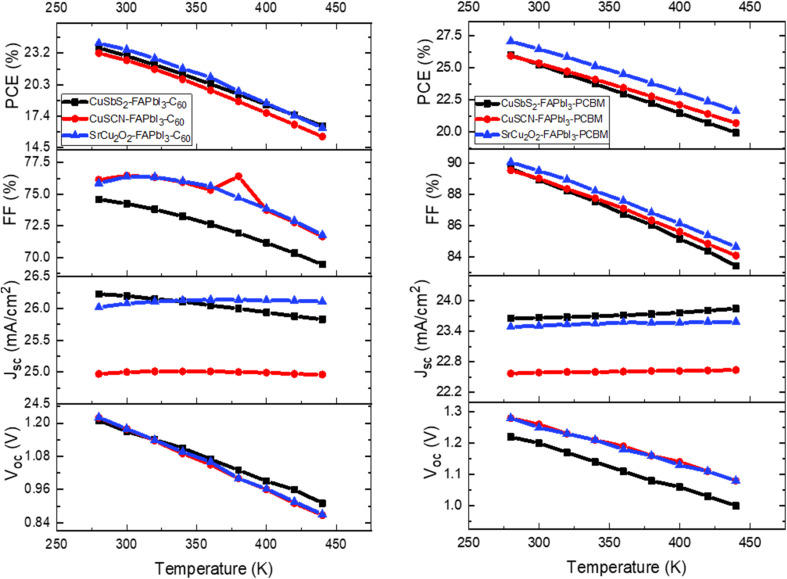
Influence of working temperature on device output characteristics.

To investigate the effect of working temperature on the solar cell performance, the temperature is varied from 280 to 400 K for all the proposed structures. All other parameters including absorber thickness, doping concentration and CTL thickness are set to their optimized values given in Table 3. From the results of Fig. 12 it is observed that as working temperature is increased the output performance of the structures reduced drastically. This occurs because series resistance (*R*_S_) increases with the rise in temperature which incites reduction in diffusion length and ultimately leads to increase in recombination rate. This trend is consistent with prior research on temperature-dependent solar cell performance.^42^ The recommended temperature of the simulated model is fixed at 300 K to attain improved cell efficiency.

### 3.10 Effect of interface defects

**Fig. 13:**
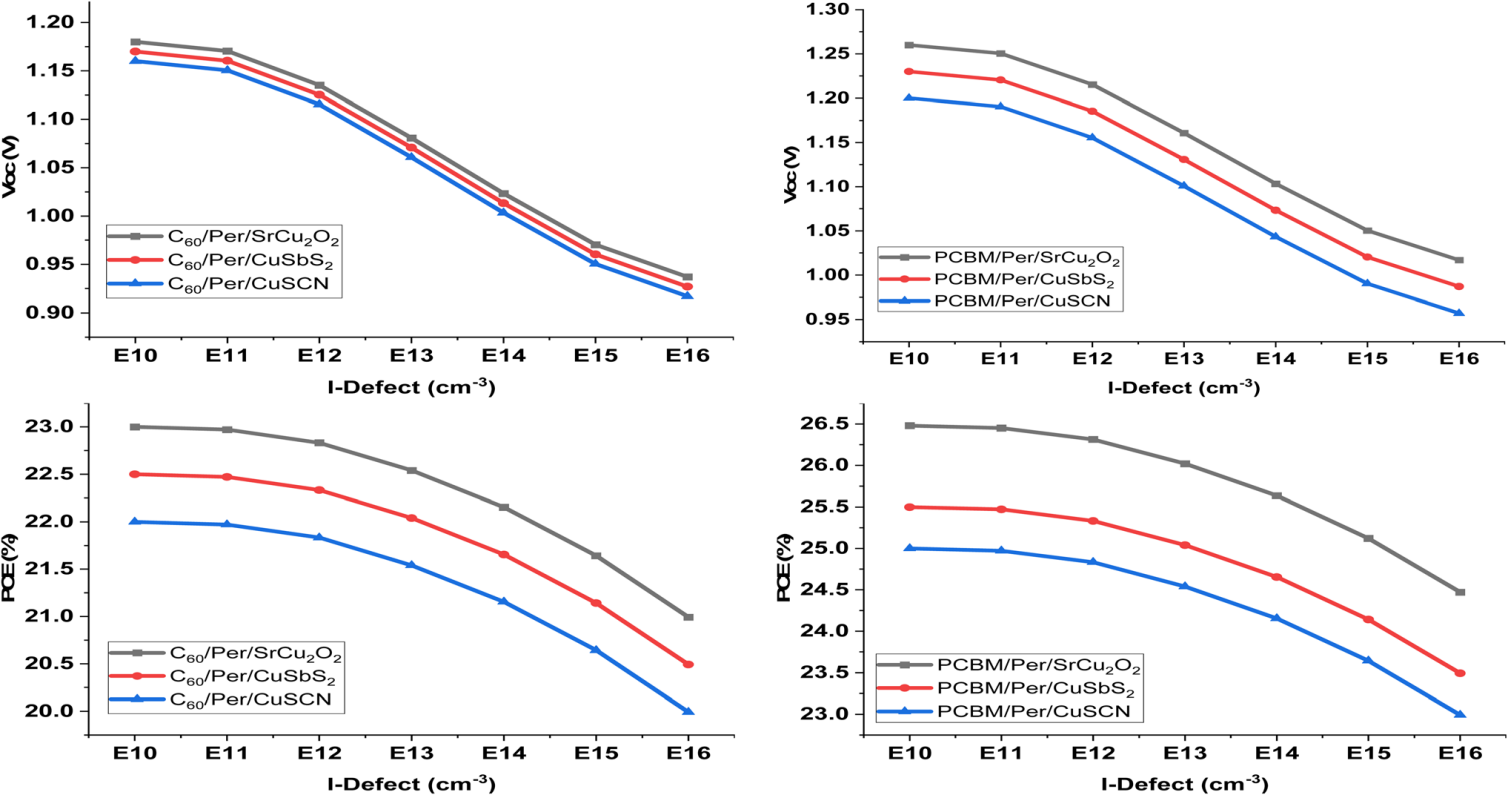
Influence of HTL/perovskite defects on device output characteristics.

**Fig. 14:**
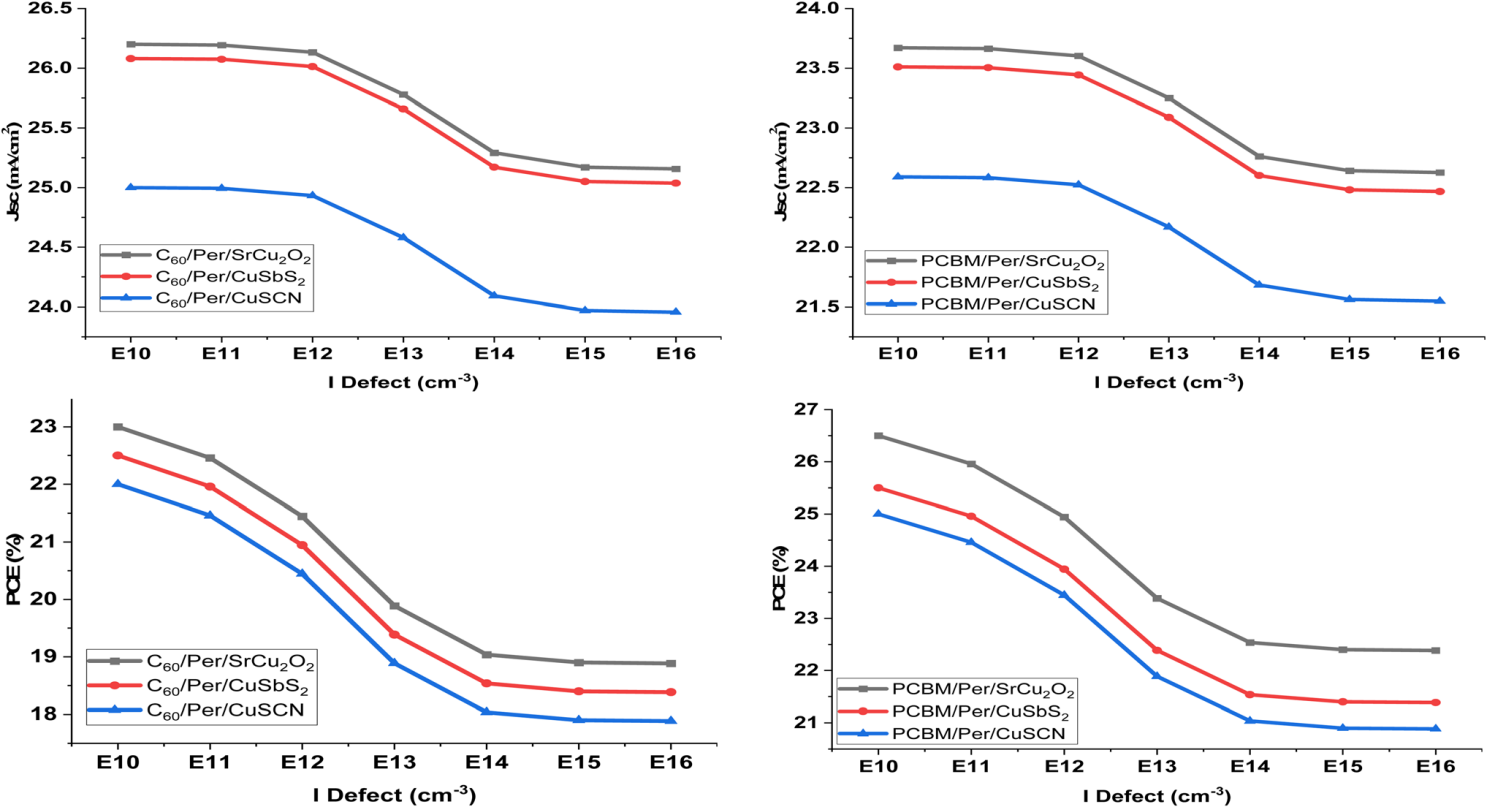
Influence of ETL/perovskite defects on device output characteristics.

The perovskite solar cell has two main interfaces. The first is the ETL/perovskite interface while the second is the HTL/perovskite interface. The defects at the interface occur due to the dangling bonds and grain boundaries formed at the surfaces of the materials. These act as traps for the charge carriers and capture them when they are being transported to the CTL from the perovskite material. To study the effect of interface defects on the PSC performance, both interface defects are changed from 1 × 10^10^ to 1 × 10^16^ cm^−3^. Fig. 13 show the effect of HTL/perovskite interface defect on PSC performance while Fig. 14 show the effect of perovskite/ETL interface defects.

The HTL/perovskite interface reduces the *V*_OC_ of all the structures as the defect are increased. This happens because due to the increasing number of interface defects more traps are produced which capture more and more holes. While the ETL/perovskite interface reduces the *J*_SC_ of the PSC as the defect are increased because a greater number of electrons are captured in the traps. Both the interface defects reduce the performance of the PSC.

## 4 Conclusion

In this work, formamidinium lead tri-iodide (FAPbI_3_) is investigated with three copper based HTLs (SrCu_2_O_2_, CuSCN, CuSbS_2_) and two carbon based ETLs (C_60_ and PCBM) using SCAPS 1-D simulation software. Each CTL is observed separately by using possible combination with FAPbI_3_ absorber layer to identify optimum values for each layer. Optimum thickness and doping concentration for all the layers of the different structures are found by adopting a systematic study. It was concluded from the study that by using optimized values for each structure the best cell output performance is observed. Moreover, it is also observed that increase in doping density of ETL and HTL boots up the cell performance. Among all the structures, SrCu_2_O_2_/FAPbI_3_/PCBM showed the most efficient and promising results having PCE of 26.48% with 23.52 mA cm^−2^*J*_SC_, 1.25 V *V*_OC_ and 89.5% FF. The optimized thickness is observed at 750 nm, 150 nm and 100 nm for the absorber layer, HTL and ETL respectively. While the optimized doping concentration is observed at 1 × 10^18^ and 1 × 10^19^ for the absorber and CTLs respectively.

## Data availability

The datasets generated and analyzed during the current study are available from the corresponding author on reasonable request.

## Conflicts of interest

There are no conflicts to declare.

## Supplementary Material
